# Multivariate methods and software for association mapping in dose‐response genome‐wide association studies

**DOI:** 10.1186/1756-0381-5-21

**Published:** 2012-12-12

**Authors:** Chad C Brown, Tammy M Havener, Marisa Wong Medina, Ronald M Krauss, Howard L McLeod, Alison A Motsinger‐Reif

**Affiliations:** 1 Department of Statistics, North Carolina State University, Raleigh, NC, USA; 2 Institute for Pharmacogenomics and Individualized Therapy, University of North Carolina at Chapel Hill, Chapel Hill, NC, USA; 3 Bioinformatics Research Center, North Carolina State University, Raleigh, NC, USA; 4, Children’s Hospital Oakland Research Institute, Oakland, CA 94609, USA

**Keywords:** Pharmacogenomics, Lymphoblastoid cell lines, Chemosensitivity, Chemotherapy, Temozolomide, Idarubicin, MANOVA, GWAS, Simulation study

## Abstract

**Background:**

The large sample sizes, freedom of ethical restrictions and ease of repeated measurements make cytotoxicity assays of immortalized lymphoblastoid cell lines a powerful new *in vitro* method in pharmacogenomics research. However, previous studies may have over‐simplified the complex differences in dose‐response profiles between genotypes, resulting in a loss of power.

**Methods:**

The current study investigates four previously studied methods, plus one new method based on a multivariate analysis of variance (MANOVA) design. A simulation study was performed using differences in cancer drug response between genotypes for biologically meaningful loci. These loci also showed significance in separate genome‐wide association studies. This manuscript builds upon a previous study, where differences in dose‐response curves between genotypes were constructed using the hill slope equation.

**Conclusion:**

Overall, MANOVA was found to be the most powerful method for detecting real signals, and was also the most robust method for detection using alternatives generated with the previous simulation study. This method is also attractive because test statistics follow their expected distributions under the null hypothesis for both simulated and real data. The success of this method inspired the creation of the software program MAGWAS. MAGWAS is a computationally efficient, user‐friendly, open source software tool that works on most platforms and performs GWASs for individuals having multivariate responses using standard file formats.

## Background

While genotyping technology can provide valuable individual genetic data, pharmacogenomics research has met little success in using this data to improve patient outcomes. This result is less surprising, considering that pharmacogenomics studies with human populations often map a limited *a priori* set of targeted genes for therapeutic agents whose heritability is unknown using clinical trial data with limited sample sizes and many potential confounders [1,2]. Developed in response to these limitations, a new *in vitro* assay system utilizing dose‐response (DR) profiles of immortalized lymphoblastoid cell lines (LCLs) allows for rapid experimental testing of multiple agents using virtually unlimited sample sizes, for either linkage or association at the genome‐wide level [3]‐ [13]. A more thorough discussion of the practical benefits for using LCLs in pharmacogenomics research can be found in recent review articles [1,2].

However, obstacles for using LCLs to identify clinically relevant loci include elucidation of the translational relevance between donor and LCL, and the development of methods that are powerful for using DR data in gene mapping. For this second item, gene mapping studies (such as in genome‐wide association studies, or GWASs) attempt to find loci with genotypes having significantly different phenotypes. For cell models and other DR data, phenotypes are complex, and “different” is not well defined. Previous simulation studies show that the most powerful method depends on what these differences are [14]. For example, if differences in the DR curves between genotypes are due entirely to half inhibitory concentration (IC50), then summarizing each curve with it’s estimated IC50 and treating that as the response is the best course of action. However, if the true differences between genotypes is not IC50, then this method may not perform optimally. We would like a method that is robust to differences in the dose‐response profiles between genotypes. We would also like a method that is very powerful at detecting those kinds of differences that are likely to arise from biology. For instance, if a certain polymorphism affects the expression of an enzyme involved with inactivating a drug target, what kinds of differences in the DR profiles for LCL cytotoxicity will this polymorphism produce? Many previous studies have fit a non‐linear equation (often without testing the appropriateness of this model) to each DR curve, choosing one parameter from this fit, and perform association using this parameter estimate [3]‐ [13]. As mentioned above, the “true” differences in the DR profiles between phenotypes may not be captured by this parameter.

A previous study investigated using many different univariate summaries (such as parameter estimates from a hill slope fit) as the response in simulated association testing [14]. This study investigated the power each summary measure had in association testing, using many different simulated differences in the DR profiles between genotypes. Every summary measure performed poorly under at least one alternative. However, methods that modeled all of the responses jointly performed at least adequately (and sometimes very well) under every alternative. At question, however, is which alternative(s) is(are) most representative of the differences between in the DR profiles between genotypes for meaningful loci that are likely to be encountered in practice? To this end, the current study uses actual differences in curves between genotypes from real data for effects that have strong support for representing true biology. These differences will be referred to as *signals* throughout. Each of these signals was created from markers that were found to be significant in genetic association studies and also had been validated from other data or from previous results. In this way, using real data to guide a simulation study, where these differences can be modeled after genuine biological signals, may give a more accurate assessment of which method performs best in practice.

## Methods

### Biological motivation for simulation study

The signals used for study were all from a set of GWASs of 515 LCLs exposed to either the cancer drug temozolomide or 5‐fluorouracil. A strong association (p= 3.3∗10^−16^) was found between LCL cytotoxicity to temozolomide and locus *rs531572*, located within the gene coding for *O*^6^*‐methylguanine‐DNA methyltransferase* [*MGMT*: ENSG00000170430]. In addition, a strong association (p= 6.8∗10^−26^) was found between the same loci and expression levels for MGMT transcripts [15]. *MGMT* is known to repair DNA damaged by temozolomide, and genetic variants affecting *MGMT* are known to be associated with temozolomide clinical efficacy [16]. Similarly, suggestive associations (*p*= 5.9∗10^−7^ and) were found between LCL cytotoxicity to 5‐fluorouracil and locus *rs2270311*, located within the gene coding for chimerin 2 [*CHN2*: ENSG00000106069]. Significant differences in the expression of CHN2 have been found between between colon cancer cells having different levels of 5‐fluorouracil resistance [17].

**Figure 1:**
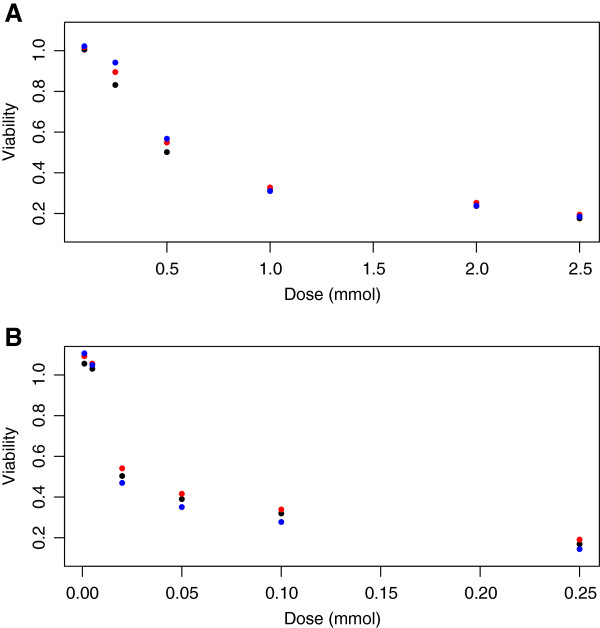
**Real signal based on temozolomide and 5‐fluorouracil response.** Differences in response between genotypes for **A** temozolomide at *rs531572* and **B** 5‐fluorouracil at *rs2270311*. Black, red and blue dots indicate the genotypes for 0, 1 and 2 copies of the minor allele.

Figure 1 illustrates the differences in mean viabilities between genotypes at each concentration for temozolomide and 5‐fluorouracil. The mean viability was corrected for potentially confounding covariates by least squares regression and estimation at the sample means for each covariate. These covariates include cellular growth rate, laboratory temperature, the first two genetic principal components and laboratory date (nominal). Signals are due to single nucleotide polymorphisms (SNPs), where black, red and blue circles represent genotypes for 0, 1 and 2 minor alleles, respectively.

**Figure 2:**
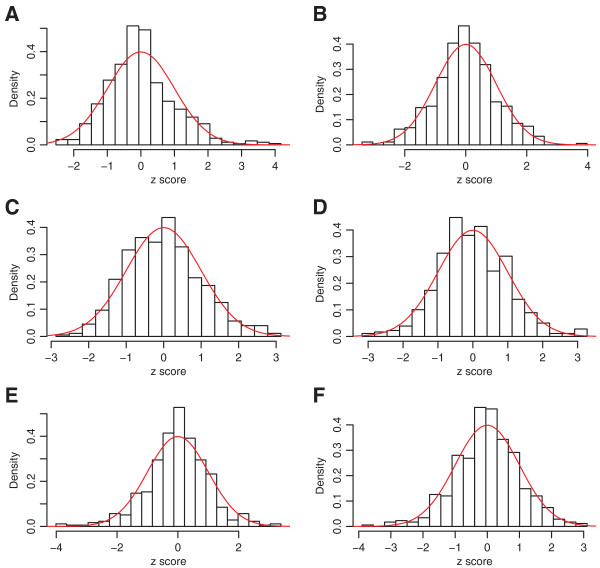
**Histograms for temozolomide at *rs531572 *.** Histogram of residuals for temozolomide at *rs531572*, with standard normal densities overlaid. Panels **A** ‐ **F** are for transformed residuals for highest to lowest drug concentrations.

**Figure 3:**
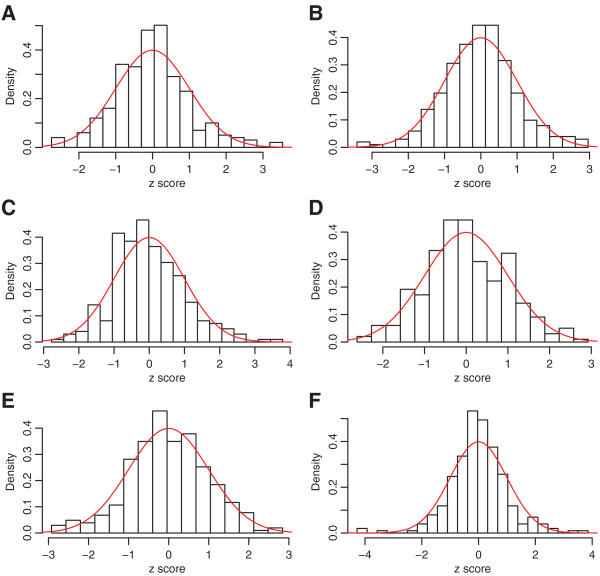
**Histograms for 5‐fluorouracil at *rs2270311 *.** Histogram of residuals for 5‐fluorouracil at *rs2270311*, with standard normal densities overlaid. Panels **A** ‐ **F** are for transformed residuals for highest to lowest drug concentrations.

**Figure 4:**
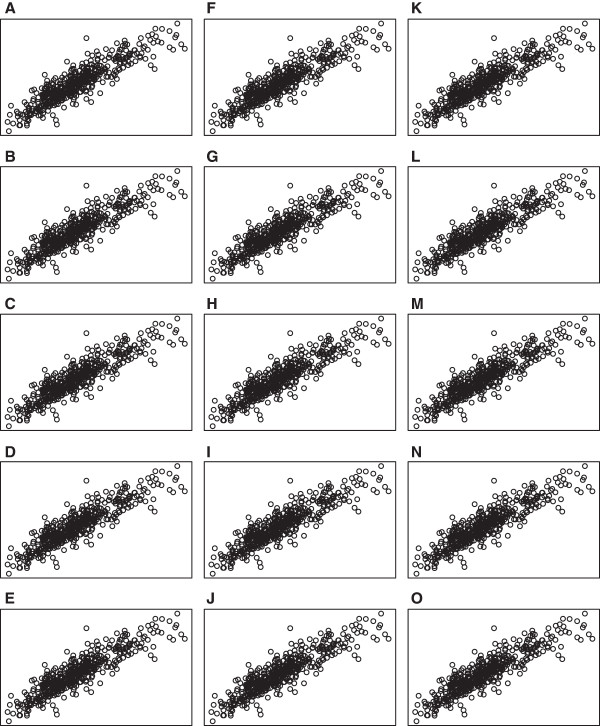
**Scatter plots for temozolomide at *rs531572 *.** Scatter plots of residual vectors between each pair of drug concentrations for temozolomide. Plots **A** ‐ **O** represent residuals between drug concentrations 1 and 2, 1 and 3, …, 5 and 6.

**Figure 5:**
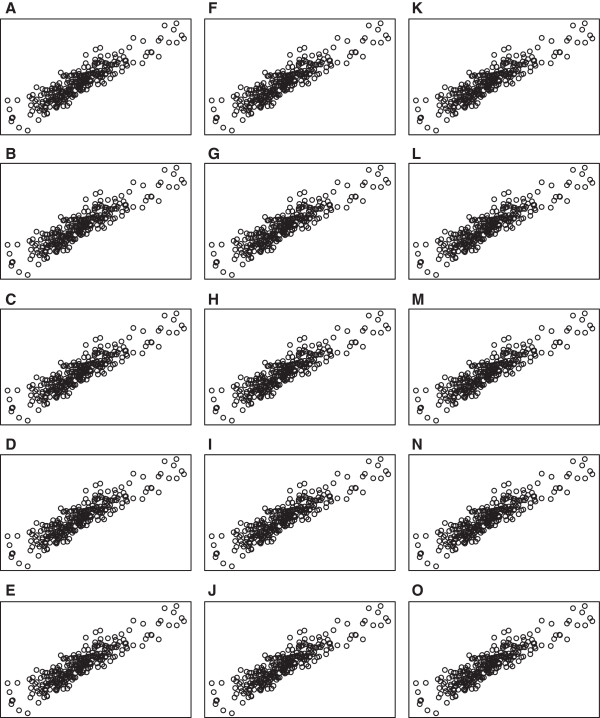
**Scatter plots for 5‐fluorouracil at *rs2270311 *.** Scatter plots of residual vectors between each pair of drug concentrations for 5‐fluorouracil. Plots **A** ‐ **O** represent residuals between drug concentrations 1 and 2, 1 and 3, …, 5 and 6.

After performing regression using the covariates mentioned above, error terms were assessed for multivariate normality. Although the error terms failed the Shapiro‐Wilk test for multivariate normality (p= 2.8∗10^−4^ for temozolomide and p= 1.4∗10^−8^ for 5‐fluorouracil, [18]), the goal of this simulation was to use real data as a guide in simulation. To this end, residuals were first transformed to be standardized and uncorrelated, according to [19]. Then histograms of errors for each drug concentration were overlaid with standard normal densities in Figures 2 and 3. In addition, Figures 4 and 5 show scatter plots of residuals between each pair of drug concentrations for temozolomide and 5‐fluorouracil. Although the distribution of errors are definitely not normal, from these plots it appears, at least visually, that the deviations from normality (with the exception of a few outliers) are not severe.

### Simulation and power comparisons of cell line methods

A simulation study was performed using the appropriate estimated means and error covariances, as described in the previous section. Using parameter estimates from these biological signals, data were generated as multivariate normal according to:

(1) $$\;\begin{array}{ccc} \;Y_{\text{ijk}} & \;∼ & N_{6}({\tilde{\mu }}_{i},\hat{\Sigma })\\ \;{\tilde{\mu }}_{i} & \;= & {\hat{\mu }}_{0}+\text{ES}\left[{\hat{\beta }}_{1}I(i=1)+{\hat{\beta }}_{2}I(i=2)\right]\\ \;{\hat{\beta }}_{m} & \;= & {\hat{\mu }}_{m}-{\hat{\mu }}_{0},\;\;m\in 1,2 \end{array}$$

where *Y*_*ijk*_ is the vector of viabilities for six concentrations for the *k*^*th*^ replication of the *j*^*th*^ individual having genotype *i* (the number of minor alleles). Here, $\hat{\Sigma }$ is the sample covariance of errors, ${\hat{\mu }}_{i}$ is the vector of mean viabilities for genotype *i*, and *ES* is the effect size. Effect sizes ranged from 0 (corresponding to the null) to 1.0 (the observed differences between genotypes). The sample size was set to 500 and minor allele frequency (MAF) was set to 0.5.

**Figure 6:**
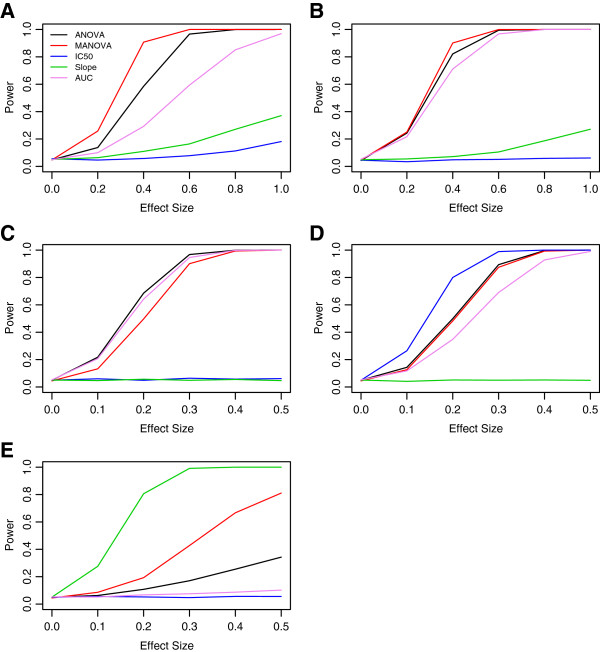
**Power curves for real and contrived alternatives.** Panels **A** and **B** are real signals based on LCL response to temozolomide at *rs531572* (within the gene MGMT) and 5‐fluorouracil at *rs2270311* (located within the gene CHN2), respectively. Panels **C**, **D** and **E** are for differences between genotypes being due to the hill slope parameters, “Min”, “IC50” and “Slope” from [14].

For each effect size and signal, 2500 data sets were used to calculate test statistics for four previously reported methods (*IC*50, *Slope*, *AUC*_*Emp*_ and *ANOVA*) [14], as well as a new method using Pillai’s trace from a multivariate analysis of variance (MANOVA) [20]. In addition, 10,000 data sets were created with an effect size of zero, and a different random number seed, to represent the null distribution. In this way, *p*‐values for each test statistic under the alternative distribution were estimated by the proportion of larger statistics under the null distribution, as described in [14]. This was required, for the *ANOVA* method, as applied to all (non‐independent) observations, generated test statistics that did not follow the expected distribution under the null. Power curves describing the proportion of times the null hypothesis of no difference between genotypes was rejected, at the alpha = 0.05 level, are illustrated in Figure 6, where panels **A** and **B** represent the power curves for simulation using signals from temozolomide/MGMT and 5‐fluorouracil/CHN2.

In addition, each of these same methods were compared using a previous simulation described in [14], where differences in the DR curves between genotypes are due to differences in the distribution of hill slope parameters. Figure 6 gives power curves for a representative sample of these simulations, where data were simulated under an additive genetic model, with equally spaced drug dosages and a MAF of 0.5. Panels **C** ‐ **E** represent power curves for each method where differences in curves between genotypes are due to the “Min”, “IC50” and “Slope” parameter distributions, respectively. Using the Friedman test, significant differences (*p* < 10^−15^ for all) in *p*‐values were found between methods for every positive (*i.e.* non‐null) effect size for both sets of simulations.

**Figure 7:**
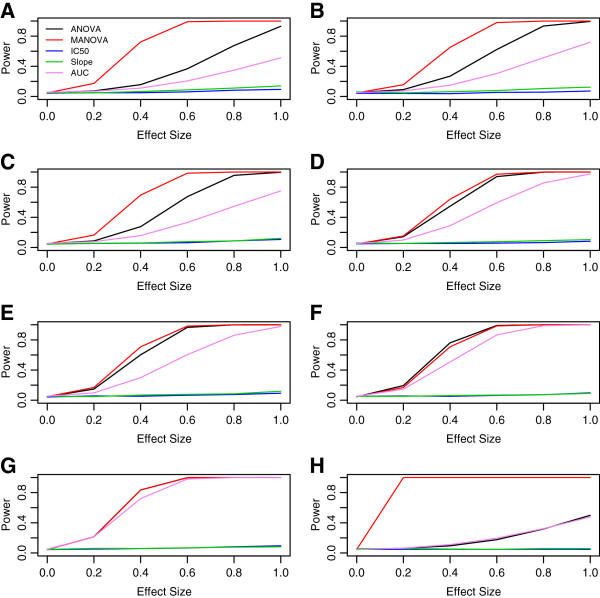
**Power curves for various error structures.** Power plots using the signal from temozolomide/MGMT with different error structures are shown in all panels. Panels **A**, **C** and **E** are under equal correlation with *ρ* = 0.75, 0.5 and 0.25, respectively. Panels **B**, **D** and **F** are under exponential correlation with *ρ* = 0.75, 0.5 and 0.25, respectively. Panels **G** and **H** had error terms generated independently as normal and gamma, respectively.

Also, the effect of modifying the error structures from Equation 1 were explored. Here, the mean vectors *μ*_*i*_ across genotypes *i* were taken from the signal for temozolomide/MGMT but the covariance matrix Σ was modified to represent various contrived correlation structures. This was done to assess how sensitive the power of MANOVA was to error structures and also to the assumption of multivariate normality. The chosen error structures include equal correlations using compound symmetric (*i.e.* constant) correlation (with *ρ* = 0.25,0.5 or 0.75), autoregressive (exponential attenuation) correlation (with *ρ* = 0.25,0.5 or 0.75) and no correlation. In addition, errors were generated independently, but using a centered gamma distribution with parameters shape=8 and scale=0.125. The results for each of these simulations is shown in Figure 7.

**Figure 8:**
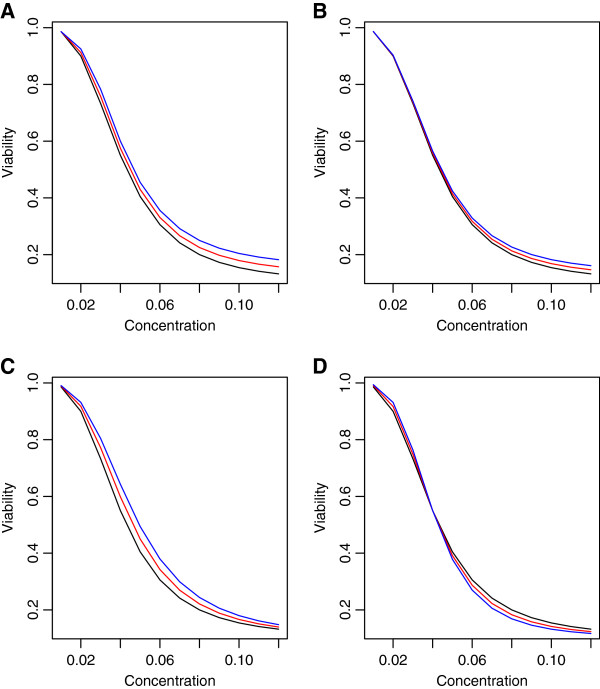
**Dose response curves for used for non‐ideal conditions.** Dose response curves used for generating data that are not ideal for MANOVA, where differences between genotypes are designed to follow univariate summary statistics. Panel **A** represents area under the curve and panels **B** ‐ **D** are for the hill slope parameters “Min”, “IC50” and “Slope”, respectively.

**Figure 9:**
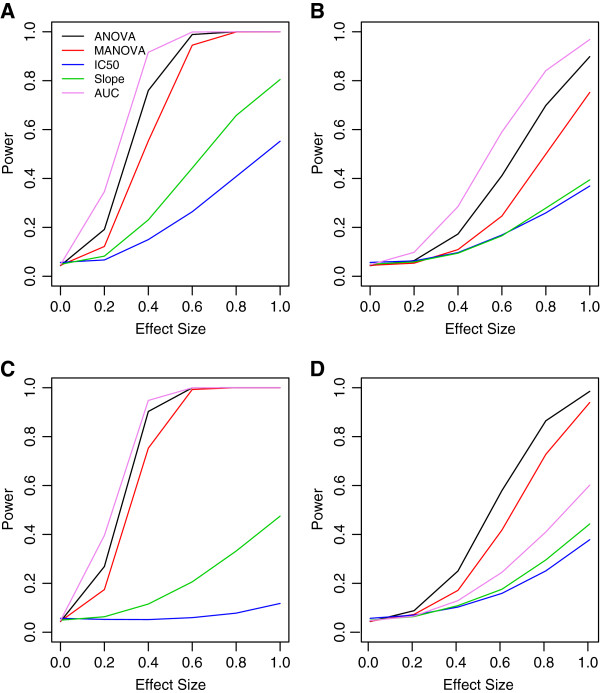
**Power curves for non‐ideal conditions.** Shown are power curves for data generated using the DR curves from Figure 8, using an exponential decay correlation structure with *ρ* = 0.25. Panels **A** ‐ **D** are for area under the curve, “Min”, “IC50” and “Slope” parameters, respectively.

Finally, simulations were performed to assess the strength of MANOVA with data generated using multivariate normal, but under non‐ideal situations. For these, mean vectors and covariances were constructed from 12 equally‐spaced doses. The difference in mean vectors between genotypes were designed to follow a specific univariate summary, including area under the curve, and the hill slope parameters “Min”, “IC50” and “Slope”, as illustrated in Figure 8. Using these mean vectors, simulations were performed where error terms follow an exponential decay correlation structure, with *ρ* = 0.25. Power plots from these simulations are illustrated in Figure 9.

## Results and discussion

The simulated data (for the current study) were generated using a multivariate normal model. In addition, MANOVA has an assumption of multivariate normality. This appears as an intentional way to provide MANOVA with an advantage, since correct modeling assumptions are guaranteed. However, our goal was actually to identify a statistical model with sufficient complexity to accurately capture most aspects of the real data. In addition, the univariate summary methods rely on ANOVA, a statistical method that is robust to the assumption of normality, reducing the inherent advantage MANOVA has over the other methods. Indeed, even though data generation was under multivariate normality, MANOVA is not assured to be the most powerful for every situation. First, if the number of responses is large, power attenuates, since the number of covariance parameters needing to be estimated grows with the square of the number of responses. Second the power of MANOVA compared to other methods seems to be reduced when the off‐diagonal covariance parameters decreases. This is shown in Figure 7, where the mean vectors for each genotype are modeled after the temozolomide and MGMT signal, but residual covariances are modeled using an exponential decay or equal correlation. Here, the dominating power of MANOVA over the other methods is reduced as the correlation between residuals decreases. A final way in which MANOVA may not perform optimally, compared with other methods, is when the differences in the DR profiles between genotypes are due entirely to a simple summary measure. These ideas are illustrated in Figures 8 and 9, where data are generated for large (12) vector responses, correlations are low (*ρ*=0.25) and differences in the DR curves between genotypes can be summarized well using simple summary measures. In this case, the MANOVA method does not perform optimally, even though data are generated using a multivariate normal model.

**Figure 10:**
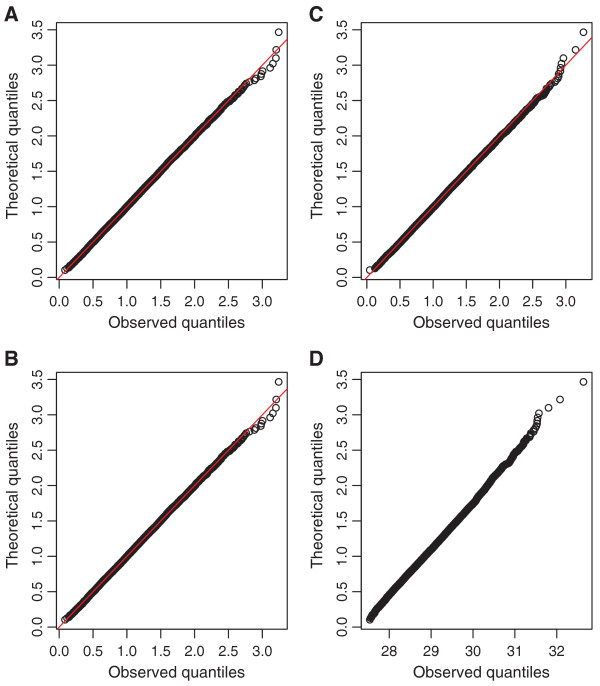
**MANOVA test statistic QQ plots.** Shown are QQ plots of MANOVA test statistics generated under the null, based on temozolomide/MGMT (panel **A**), 5‐fluorouracil/CHN2 (panel **B**), random hill slope curves from a previously reported simulation (panel **C**) [14], and error structures following a gamma distribution (panel **D**).

It is also interesting to observe how well MANOVA performs when the assumption of multivariate normality is violated. The previous simulation study relied on generating random hill slope lines, and adding error terms that follow a Laplace distribution. Even though the resulting response vectors deviate far from normal (results not shown), MANOVA still performs surprisingly well. Although never the most powerful method under these conditions, MANOVA was consistently among the most powerful, and had best performance across a range of hill slope alternatives. A few of these are illustrated in Figure 6. In addition, the advantage of MANOVA is actually increased over the other methods for the temozolomide/MGMT signal when the distribution of the error terms change from multivariate normal to gamma. These results need to be cautioned, however, because all *p*‐values were generated using resampling techniques, described in [14]. When compared to their expected distributions, MANOVA test statistics generated from the hill slope‐type simulations are well‐behaved, but the same test statistics generated with gamma distributed error terms behave quite poorly (Figure 10). This latter case would lead to inflated type‐I error rates, if *p*‐values are calculated using the expected null asymptotic distribution.

### Software implementing MANOVA in GWASs: Introducing MAGWAS

Because of the power and robustness of the MANOVA design in detecting true biological signals in LCL response, and more generally the need for analysis tools for GWASs having multivariate responses, the program MAGWAS (multivariate ANOVA genome‐wide association software) was developed [21]. MAGWAS calculates the significance of each single nucleotide polymorphisms (SNP), allows for inclusion of confounding variables and uses PLINK file formats [22,23]. The program is free, fast to download (less than 1 MB), requires no installation, is platform independent and runs using a single line on a system’s command prompt. In addition, MAGWAS is efficient with system memory and GWASs are generally completed in much less than an hour on typical desktop systems. Indeed, analysis of the two data sets for the present study where each data set had about 500 individuals, and each individual having six responses and 33 covariates, repeated across about 2 million SNPs took less than 17 minutes apiece on a modest computing cluster (two Intel(R) Xeon(R) CPU E5450 processors). MAGWAS is written completely in Java and is registered as open source software under a GNU public license. Matrix manipulations are accomplished using the Java package JAMA [24] and distributional calculations are made using the Java package DistLib [25].

## Conclusions

In conclusion, no method is uniformly superior for modeling the genetic effects for DR cell line data. However, unlike other univariate summary methods (such as estimating the IC50 for each DR curve and treating this estimate as the response), MANOVA makes no assumption about the nature of the DR curve or about what the differences in the DR curves between genotypes are expected to be. Unsurprisingly, using simulated data from a previous study, MANOVA was the only method that had good power under all alternatives considered. In addition, MANOVA was most powerful in detecting differences between genotypes for simulations based on signals obtained from real data. A final advantage of using MANOVA is that test statistics asymptotically follow a known distribution under the null, allowing for fast and accurate analysis of each loci in a GWAS, so long as sample sizes are not small. Quantile‐quantile (QQ) plots of the null distributions of test statistics from this study, as well as the null for the previous simulation, demonstrate that the type I error rate is under control in all conditions, except when data are generated using a gamma distribution for the error structure. Figure 10 shows four representative examples of these QQ plots. Under the assumption that most loci have no true association in genome‐wide data, a similar result was found for QQ plots from a separate set of GWASs (results not shown) involving LCL response to 29 different anticancer drugs.

On the other hand, MANOVA is not guaranteed to always be optimal, especially if the number of responses for each individual is large, the correlations between responses are small, and/or the true differences in DR curves between genotypes are known to be due to entirely to single univariate summary measure. Also, the interpretation for MANOVA is somewhat more complicated, as traditional measures of effect size, such as model r‐squared (R2) and parameter estimates are not immediately clear. For example, R2 may vary substantially between responses at each concentration. Similarly, parameter estimates for the effect of SNP are unlikely to be the same, and may even have opposite directions, at different concentrations. Overall, we feel the benefits outweigh the handicaps and that MANOVA is a great method for gene mapping, or other association tests utilizing DR data.

The current study explored two basic classes of linear models as methods for testing differences between genotypes for DR data. These include using ANOVA to model the differences in DR summary measures, and using MANOVA to model the differences between response vectors jointly. However, several other methods could potentially be applied for modeling DR data. One of these includes adapting methods from longitudinal and repeated measures data analysis [26]. If the basic form of the covariance structure between residuals were known, these methods could potentially reduce the number of parameters being estimated, and may be ideal, especially in situations where the number of responses (concentrations) for each individual is rather large. Another set of methods employ semi and non‐parametric designs for mapping quantitative trait loci [27]‐ [29]. Semi (non)‐parametric designs are attractive because they use minimal (no) modeling assumptions, therefore guarding against incorrect model specification. Future studies would benefit from comparing these designs to MANOVA in DR data from LCL cytotoxicities.

## Abbreviations

DR: dose response; LCL: lymphoblastoid cell line; GWAS: genome‐wide association study; QQ: quantile‐quantile; ANOVA: analysis of variance; MANOVA: multivariate ANOVA

## Competing interests

The authors declare that they have no competing interests.

## Authors’ contributions

CCB wrote the manuscript, performed all analysis and created magwas software. TMH collected LCL data. MWM and RMK provided and performed genotyping for the Epstein‐Barr virus immortalized LCLs. HLM revised the manuscript and designed the original linkage and association studies. AAM revised the manuscript and helped design the simulation and real data experiments. All authors read and approved the final manuscript.
